# Microvascular Response in Patients with Complex Regional Pain Syndrome as Measured by Laser Doppler Imaging

**DOI:** 10.1111/micc.12286

**Published:** 2016-07-10

**Authors:** Rachel Gorodkin, Ariane L. Herrick, Andrea K. Murray

**Affiliations:** ^1^ The Kellgren Centre for RheumatologyManchester Royal InfirmaryManchesterUK; ^2^ Institute of Inflammation and RepairManchester Academic Health Science CentreSalford Royal NHS Foundation TrustThe University of ManchesterSalfordUK; ^3^ NIHR Manchester Musculoskeletal Biomedical Research UnitCentral Manchester NHS Foundation TrustManchester Academic Health Science CentreManchesterUK; ^4^ Photon Science InstituteUniversity of ManchesterManchesterUK

**Keywords:** complex regional pain syndrome, blood flow, laser Doppler imaging

## Abstract

**Objectives:**

Our aim was to investigate the hypothesis that microvascular dysfunction occurs in patients with CRPS. Specifically, whether there were functional differences in either deeper cutaneous blood vessels or more superficial nutritive vessels between the affected and unaffected limb in patients with CRPS, and between CRPS patients and healthy control subjects.

**Methods:**

Twenty‐two patients with CRPS (five male; mean age 45 years; eight upper limb involvement, 14 lower limb) and 23 healthy control subjects (one male; 43 years) were recruited. Microvascular flow at affected and unaffected contralateral sites was measured, following local heating, using laser Doppler imaging (red/green wavelengths). Corresponding sites were imaged in healthy controls. Maximum flux level and area under the curve (first 20 scans, AUC20) were measured.

**Results:**

Vasodilator responses to heat were similar in affected and unaffected limbs, and in healthy controls. For example, median (IQR) “red” AUC20 in CRPS was 138.6 (120.0–152.9)% change from baseline in affected limb and 135.0 (120.7–166.8)% in unaffected limb, and (in healthy controls) 133.1 (117.2–145.9)% and 139.1% (126.0–162.1) in limb 1 and 2.

**Conclusions:**

We found no impairment of vasodilation in cutaneous microvessels in CRPS. The vasomotor changes in CRPS may relate to larger vessel dysfunction.

**Abbreviations used**
CRPS: complex regional pain syndrome
LDI: laser Doppler imaging

## Introduction

CRPS is a descriptive term used to describe the painful swelling of an extremity, usually associated with a preceding injury 2, 9. Upper or lower limb can be affected. Pain can be extreme, and affected patients are often severely distressed and disabled. The pathophysiology of CRPS is incompletely understood: peripheral and central mechanisms, neurogenic inflammation, and microvascular dysfunction have all been proposed 9. It is likely that all contribute either as initiators or perpetuators of the “vicious cycle” of CRPS, and that all inter‐relate. Current treatments are often unsatisfactory, highlighting the importance of increasing our understanding of the underlying disease mechanisms in order to inform new directions in therapy.

Vasomotor changes are well recognized in CRPS, with color or temperature changes in the affected extremity. In the most characteristic case, the affected limb is initially warm and red, becoming mottled and intermittently warm and cool before becoming more permanently cold. However, some patients report coldness of the affected limb from the outset. It remains unknown whether these vasomotor changes relate to abnormalities of the cutaneous microvasculature at the affected site. This is a relatively under‐researched area, although different investigators have reported microvascular dysfunction 13, and affected limbs have been shown to be hypoxic 8. It has been suggested that an imbalance between vasoconstriction and vasodilation occurs 3.

The aim of this study was to investigate the hypothesis that microvascular dysfunction occurs in the skin of patients with CRPS. If so, then treatments targeting microvascular pathology might be beneficial. Specifically, we sought to identify whether there were differences in either deeper cutaneous blood vessels (as measured by red LDI) or the more superficial nutritive vessels (as measured by green LDI), as discussed previously, 10 between the affected and unaffected limb in patients with CRPS, and between CRPS patients and healthy control subjects. Local heating was chosen as the most suitable physiological stimulus.

## Patients and Methods

Twenty‐two patients with CRPS (five male) and 23 healthy control subjects (one male) were recruited. The mean age of patients was 45 (range 20–77) years and of control subjects 43 (24–70) years. Mean disease duration was four (range 0.25–14) years. Six patients and one control were smokers. Thermography of patients was carried out prior to the laser Doppler protocol. Classification was based upon thermography and patient description. Six patients described their affected limb as predominantly “hot” and 14 as predominantly “cold” (although only one had an unequivocally “hot” limb as judged by clinical examination and thermography). Fourteen patients had lower limb involvement and eight had upper limb. All subjects gave written informed consent. The study had the approval of the Salford and Trafford Local Research Ethics Committee.

Subjects were acclimatized for 20 minutes in a temperature‐controlled room at 23 ± 1°C. For patients with upper limb CRPS, the dorsum of the hand was preferentially examined and for patients with lower limb CRPS, the medial border of the calf. These sites were chosen to allow logistically easy imaging and minimize discomfort to the patient. The same sites were used in the control subjects. In all cases, the bilateral position was used as an internal control.

Scans were carried out using dual wavelength LDI (red (633 nm) and green 532 nm 10; Moor Instruments Ltd., Axminster, UK and Laser Quantum, Manchester, UK).

A baseline scan was taken. A heating pad was then positioned and location marked, by dots, on the skin (Periflux PF3 pad, 3 cm in diameter; Perimed UK, Bury St Edmunds, UK). Heating commenced as follows: 34°C for 30 seconds; 36°C for 30 seconds; 38°C for three minutes. Gradual heating and relatively low maximum temperature were chosen to minimize potential discomfort (some patients with CRPS are unable to tolerate higher temperatures due to heat allodynia).

Following heating, the pad was removed and a series of scans commenced. Scans continued until the flux levels returned to baseline.

In all patients, four series of scans were collected in random order (but each series of scans always alternated from one limb to the other)—red and green laser scans for the affected limb and equivalent contralateral site. The same procedure was carried out for control subjects, with both right and left limbs (either upper or lower) being studied.

Flux images were analyzed (Moor LDI v3.08, Moor Instruments Ltd.) by taking the median blood flow (arbitrary perfusion units) of the heated area; the outline of the area selected was fitted to the dots marked on the skin. Two parameters were calculated:

1. Flux_max_ the maximum flux level
2. AUC20 the area under the flux curve for the first 20 scans. Scan series that were too short (due to a rapid return to baseline flux levels) were extended utilizing the flux level from the baseline scan.

Both parameters were expressed as a percentage change from baseline.

To allow multiple comparisons, statistical analysis was carried out using repeated‐measures analysis of variants ([ANOVA]; SPSS, Chicago, IL, USA).

## Results

**Figure 1:**
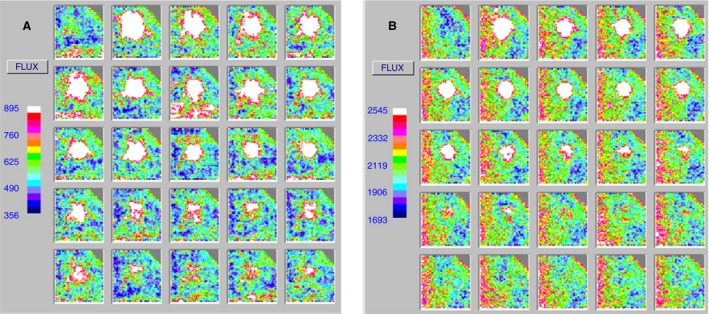
Examples of green and red LDI scans (healthy control). Typical example of LDI repeat scans from a healthy control: (**A**) green (532 nm) LDI repeat scan (**B**) red repeat (633 nm) LDI scan. Flux is measured in perfusion units, scale shown to the left of the image. The first scan (top left of each set of repeat scans) was the baseline scan; the adjacent scan to the right of this is the first post heating scan. Scans were continued until flux levels had returned to baseline.

**Figure 2:**
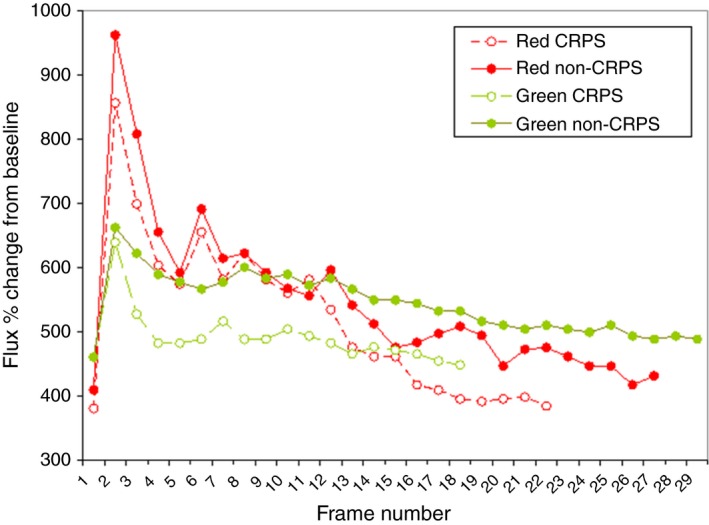
Plot of typical red and green LDI data from repeat scans following local heating of a single CRPS subject and a healthy control. For patients with upper limb CRPS, the dorsum of the hand was preferentially examined and for patients with lower limb CRPS, the medial border of the calf. The same sites were used in the control subjects. In all cases, the bilateral position was used as an internal control. A baseline scan was taken, a heating pad positioned and the area heated: 34°C for 30 seconds; 36°C for 30 seconds; 38°C for three minutes.

Patients and controls tolerated the procedure well with the exception of some mild heat‐induced allodynia in two patients. An example of repeat scans (red and green wavelengths) is shown in Figure 1A and B. Figure 2 shows plotted data of flux with time for both a patient with CRPS and a healthy control.

### Flux_max_

#### Red LDI

The median (interquartile range [IQR]) flux_max_ in CRPS was 266.9% (195.1–410.8) in affected limb and 252.6% (209.0–252.6) in unaffected limb, and (in healthy controls) 318.6% (231.7–344.0) in limb 1 and 336.7% (275.2–387.5) in limb 2.

#### Green LDI

The median (IQR) flux_max_ in CRPS was 130.9% (118.4–155.6) in affected limb and 126.1% (114.3–151.8) in unaffected limb, and (in healthy controls) 144.4% (130.6–166.6) in limb 1 and 146.7% (133.5–163.5) in limb 2.

Repeated measures ANOVA taking the laser wavelength into account showed no greater difference between affected and unaffected limbs than between the two control limbs (*p* = 0.294). The difference between the CRPS and control groups was also nonsignificant (*p* = 0.347).

### AUC20

#### Red LDI

The median (IQR) AUC20 in CRPS was 138.6% (120.0–152.9) in affected limb and 135.0% (120.7–166.8) in unaffected limb, and (in healthy controls) 133.1% (117.2–145.9) in limb 1 and 139.1% (126.0–162.1) in limb 2.

#### Green LDI

The median (IQR) AUC20 in CRPS was 105.3% (101.2–111.5) in affected limb and 105.4% (101.6–111.5) in unaffected limb, and (in healthy controls) 106.6% (103.9–113.2) in limb 1 and 108.0 (103.3–112.9) in limb 2.

Again, there was no greater difference between affected and unaffected limbs than between the two control limbs (*p* = 0.505) and the difference between the CRPS and control groups was nonsignificant (*p* = 0.752).

## Discussion

The novelty of our study was the examination of both superficial (nutritive) and deeper microvessels in the skin of patients with CRPS by dual wavelength LDI. LDI provides a non‐invasive and “non‐contact” measure of skin blood flow and is therefore an ideal technique to study a condition where the microvasculature is likely to be abnormal, but where hyperalgesia and allodynia also play a significant role.

We found no differences in microvascular responses in affected compared to unaffected limbs, nor between patients with CRPS and healthy controls. These findings are therefore consistent with our earlier study, which suggested no impairment in microvascular endothelial function in CRPS, assessed using iontophoresis of the endothelial‐dependent vasodilator acetylcholine chloride, and the endothelial‐independent vasodilator sodium nitroprusside 5. In contrast, Schattschneider *et al*. 11 did find impairment of endothelial‐dependent (but not endothelial‐independent) vasodilation in CRPS, although patients all had “chronic cold” CRPS which might explain the difference.

Our study demonstrated the inherent difficulties and challenges of clinical studies in patients with CRPS. Comparing the contralateral sites allowed an internal “control” measurement, matched in terms of baseline values. However, since CRPS may cause systemic changes in skin microvascular function, data were also taken in a set of healthy controls. Essentially results were “negative,” but as with so many previous studies of CRPS we do not know whether this was a true result (i.e., cutaneous microvascular responses are normal in CRPS) or whether the heterogeneity and relative rarity of the condition (resulting in small numbers of patients being studied) limit the conclusions that can be drawn. While we accept that the small number of patients included was a limitation of our study, numbers were comparable to those in other physiological studies of CRPS. We minimized the potential variation in blood flow between different anatomical sites by comparing bilateral sites within patients.

Our study has confirmed the feasibility of LDI for studying microvascular pathophysiology in CRPS, but highlights the need for larger, multicenter studies, allowing subgroup analysis. Patients with “hot” and “cold” limbs likely behave very differently and it is probable that the mechanism for “cold” and “hot” CRPS differs. Both classifications of patients were included since it was of interest to see whether “hot” and “cold” limbed patients had the same response to the heating protocol. Clinical features vary considerably over time, given the large duration differences between patients in this study, it is possible that some patients were in an acute phase, others in a chronic phase. Ideally prospective longitudinal studies are required to examine change over time. LDI has been previously applied in only a small number of CRPS studies; to monitor axon flare response to repetitive noxious electrical stimuli, with contrasting results (increased perfusion in patients compared to healthy controls 14 and no difference 12), and to identify abnormal perfusion in patients with recurrent postoperative CRPS 1.

### Future Therapeutic Strategies

Resolving the contribution of local blood flow impairment, leading to tissue hypoxia and free‐radical mediated injury, in CRPS pathogenesis, may identify new therapeutic strategies. Evidence suggests that vitamin C, an antioxidant, may prevent CRPS 15, and free radical scavengers may be effective in treatment 4. The phosphodiesterase inhibitor tadalafil (a vasodilator) reduced pain in “cold” CRPS 6 although conversely topically applied isosorbide dinitrate (a nitric oxide donor) did not confer benefit 7.

## Perspective

In conclusion, we did not find impaired vasodilation in patients with CRPS, although the small patient numbers and heterogeneity of the disease (“hot” and “cold,” upper and lower limb involvement) limit our conclusions. Larger studies would allow subanalysis of upper and lower limb and the differences between “hot” and “cold” CRPS to further understanding. Vasomotor changes in CRPS may relate to large vessel dysfunction, or occur primarily in subgroups of patients not well represented in this small study.

## Funding

This work was supported by Arthritis Research UK (grant reference G0570). A.K.M. is funded by an Arthritis Research UK Career Development Fellowship. Grant number (19465).

## Conflict of Interest

The authors declare no conflicts of interest.
